# Mechanistic Insights into the Anti-Hepatocellular Carcinoma Effects of ACY-1215: p53 Acetylation and Ubiquitination Regulation

**DOI:** 10.3390/cimb47050338

**Published:** 2025-05-08

**Authors:** Yi Yin, Yutong Du, Yiting Xu, Zhuan Zhu, Yu Hu, Lingling Xu, Kunming Yang, Tian Chen, Yuyang Shi, Chengcheng Wang, Yali Zhang

**Affiliations:** South Campus, Medical College of Guizhou University, Guiyang 550025, China; yinyi200009@163.com (Y.Y.); dyt630876889@163.com (Y.D.); xuyiting0321@163.com (Y.X.); 18386569021@163.com (Z.Z.); m17375713903@163.com (Y.H.); 19855499047@163.com (L.X.); 17503433818@163.com (K.Y.); chanadd11@163.com (T.C.); 18849665237@163.com (Y.S.)

**Keywords:** hepatocellular carcinoma, p53, histone deacetylase 6, MDM2 protein, acetylation, ubiquitination

## Abstract

As a major global health challenge, hepatocellular carcinoma (HCC) still faces substantial limitations in its treatment options. This study investigates the anti-HCC potential of ACY-1215, a selective Histone deacetylase 6 (HDAC6) inhibitor, and its mechanism targeting p53 regulation. In vitro studies conducted with HepG2 and SMMC-7721 cells revealed that ACY-1215 markedly inhibited HCC cell proliferation, migratory capacity, and invasive potential, as evidenced by CCK-8, colony formation, and Transwell assays. Furthermore, ACY-1215 induced caspase-dependent apoptosis. Mechanistically, ACY-1215 enhanced p53 acetylation by disrupting HDAC6-p53 interaction, thereby stabilizing p53 protein levels. Concurrently, it inhibited Murine Double Minute 2 (MDM2)-mediated ubiquitination, blocking proteasomal degradation and prolonging p53 half-life. This dual modulation restored p53 transcriptional activity, leading to the upregulation of downstream effector molecules associated with cell cycle regulation and apoptosis. Collectively, our findings reveal that ACY-1215 exerts potent anti-HCC effects through coordinated regulation of p53 acetylation and ubiquitination, offering a novel dual-targeting strategy for HCC therapy.

## 1. Introduction

Hepatocellular carcinoma, the most prevalent form of primary liver cancer, ranks as the sixth most common malignancy worldwide and the third leading cause of cancer-associated mortality, representing a critical global health burden [1,2]. The main risk factors for HCC include infection with hepatitis viruses, fatty liver, liver cirrhosis, alcohol abuse, and other metabolic diseases [3]. Early-stage liver cancer patients have a good prognosis after undergoing hepatectomy, but due to the lack of typical clinical symptoms in the early stages, about 70% of patients are diagnosed at a terminal stage [4]. HCC exhibits a high degree of heterogeneity both at the molecular level and in histological characteristics, which further complicates clinical diagnosis and treatment. Currently, patients with terminal HCC are primarily treated with a combination of radiotherapy, chemotherapy, and targeted therapy [5]. Among these, systemic targeted therapy plays a crucial role and is mainly divided into two categories: multi-kinase inhibitors (MKIs) and immune checkpoint inhibitors [6]. Sorafenib, as a representative of multi-target kinase inhibitors, extends median survival time by 3 to 5 months in patients. However, it is accompanied by severe adverse effects and a high rate of resistance development [7]. This treatment dilemma underscores the pressing need for the exploration of novel therapeutic targets.

Research suggests that activation of oncogenes and inactivation of tumor suppressor genes play a critical role in the development of HCC [8]. As a key tumor suppressor, the p53 protein plays a central role in maintaining genomic stability through its unique molecular regulatory network. The protein consists of 393 amino acids and its functional activity is precisely regulated by dynamic phosphorylation modifications [9]. Although mutations in the p53 gene are seen in more than 50% of human malignant tumors, the prevalence of mutant p53 in HCC is significantly lower than that of other solid tumors (about 20–35%) [10]. Wild-type p53 (wt-p53), by activating downstream target genes such as p21, Bax, and PTEN, demonstrates multifaceted antitumor effects in inhibiting angiogenesis, halting cell cycle progression, inducing cellular senescence and apoptosis [11]. In the absence of genetic mutations, p53 signaling pathway’s epigenetic disarray (such as abnormal post-translational modifications) can contribute to the malignant progression of HCC [12].

The p53 protein contains multiple evolutionarily conserved functional domains whose activity is dynamically regulated by post-translational modifications such as phosphorylation, ubiquitination, and acetylation [13]. Among these, acetylation modification not only bears close relation to protein stability but also serves as a key molecular switch to activate its tumor suppressor function [14]. This modification process is governed by histone acetyltransferases (HATs) and histone deacetylases (HDACs). Histone deacetylase 6 (HDAC6), belonging to the IIb class HDAC family, mainly localizes in the cytoplasm and exhibits overexpression characteristics in various malignant tumors, including lung cancer and melanoma [15]. It was confirmed by gene ablation assay that HDAC6 acts directly on the K320 site of p53 through its deacetylase activity to maintain its hypoacetylated state. In the livers of HDAC6 knockout mice, K320 acetylation was significantly accumulated, suggesting that HDAC6 is the major deacetylase at this site [16]. Additionally, MDM2, as a classical p53-specific E3 ubiquitin ligase, mediates its proteasome-dependent degradation [17].

ACY-1215 is the first orally bioavailable selective HDAC6 inhibitor and has shown sufficient efficacy in the treatment of a variety of tumors [18]. Early studies have shown that ACY-1215 prevents non-alcoholic liver disease [19] and acute liver failure [20]. However, its effects on hepatocellular carcinoma have not been investigated.

In this study, we identified a novel mechanism by which ACY-1215 regulates p53 stability. Specifically, ACY-1215 modulates p53 acetylation mediated by HDAC6 and p53 ubiquitination mediated by MDM2. ACY-1215 not only exhibited potent anti-hepatocellular carcinoma activity, but also may provide a new way to restore the oncogenic function of p53 in hepatocellular carcinoma.

## 2. Materials and Methods

### 2.1. Chemicals and Reagents

ACY-1215 (Yuanye Bio-Technology Co., Ltd., Shanghai, China) stock solutions were dissolved in DMSO. RPMI 1640 medium and minimum essential medium (MEM) were purchased from BasalMedia (Shanghai, China). E-64, chloroquine, and cycloheximide (CHX) were acquired from MCE (Monmouth Junction, NJ, USA). The fetal bovine serum (FBS) used in experimental preparations was supplied by Procell Life Science & Technology Co., Ltd. (Wuhan, China). Monoclonal immunoglobulins targeting β-actin and GAPDH were sourced from Proteintech Group, Inc. located in Wuhan, China. Immunoreagents specific for apoptotic markers (Caspase-3, PARP, Caspase-9), regulatory proteins (Bax, Bcl-2), and HDAC6 were purchased from Cell Signaling Technology, Inc. based in Boston, MA, USA. Detection reagents including HRP-conjugated goat antibodies against rabbit and mouse IgG were also provided by this supplier. Primary antibodies for p53 pathway analysis (MDM2, Ubiquitin) and post-translational modifications (Ac-lysine) were acquired from Santa Cruz Biotechnology, Inc. in Boston, MA, USA.

### 2.2. Cell Lines and Cell Culture

The HepG2 cellular line, characterized by wt-p53 expression in human liver carcinoma systems, was sourced from the Cell Bank of Chinese Academy of Sciences (Shanghai, China). SMMC-7721 hepatoma cells were procured through Hunan Fenghui Biotechnology Co. (Wuhan, China). All cell populations were maintained at 37 °C under 5% CO_2_ atmosphere using either MEM or RPMI 1640 basal media formulations containing 10% fetal bovine serum and 1% penicillin-streptomycin antibiotic cocktail.

### 2.3. CCK-8 Assay

A cellular suspension containing 4 × 10^3^ cells per well was dispensed into 96-well plates (100 μL medium volume/well) for 24-h stabilization. Following pharmacological treatment at specified concentrations, cellular systems were maintained under standard culture conditions (37 °C) for designated durations (48/72 h). Post-treatment incubation proceeded for 60 min after supplementation with CCK-8 detection reagent. Quantitative absorbance measurements at 450 nm wavelength were recorded using a Bio-Rad microplate analysis system (Bio-Rad Laboratories Co., Ltd., CA, USA). The normalized viability percentage was derived computationally through the formula:

Viability index = [(Experimental group − Blank control)/(Untreated control − Blank control)] × 100%

### 2.4. Colony Formation Assay

Cellular suspensions containing 1 × 10^3^ HepG2/SMMC-7721 cells per well were plated into six-well culture dishes (2 mL/well) and allowed to adhere for 16 h under standard conditions. Following 24-h pharmacological exposure to ACY-1215, refreshment with drug-free complete medium was performed every 72 h throughout the 14-day colony formation period. Resultant cellular aggregates were immobilized in 4% paraformaldehyde solution for 20 min, subjected to 0.1% crystal violet staining for 15 min, and digitally documented using high-resolution imaging systems.

### 2.5. Transwell Migration and Invasion Assay

In invasion analysis, 2 × 10^4^ cells suspended in serum-free medium (200 μL) were loaded into extracellular matrix-coated chambers (Corning #354234) with 8 μm porous membranes (Labselect #14341). The lower chamber contained 800 μL complete medium with 10% FBS. After 24h culture (37 °C/5% CO_2_), non-migratory cells on the upper chamber were cleared using sterile swabs. Transmigrated cells underwent 4% paraformaldehyde fixation (15 min) and 0.1% crystal violet staining (10 min). Three randomly selected microscopic fields per sample were captured using an Olympus phase contrast system (Tokyo, Japan) for ImageJ (version 1.52) quantification. Migration studies followed identical protocols excluding matrix coating.

### 2.6. Immunoblotting Analysis

Protein samples were separated on polyvinylidene difluoride membranes using sodium dodecyl sulfate-polyacrylamide gel electrophoresis (SDS-PAGE). Membranes were closed in TBST with 10% skimmed milk for 1 h and then incubated with a primary antibody for 2 h followed by 1 h of incubation with a secondary antibody. Protein detection was performed using an enhanced chemiluminescence system (Tanon, Shanghai, China).

### 2.7. Quantitative Reverse Transcription PCR

Total RNA extraction was conducted using TRIZOL reagent (Invitrogen), followed by cDNA synthesis using the Quantscript RT kit (Tiangen), with all procedures performed in accordance with the manufacturers’ protocols. Quantitative real-time PCR analysis was subsequently carried out utilizing the CFX96 Real-Time PCR detection system (Bio-Rad). The primers for *p53* amplification are 5′-CTGGCCCCTGTCATCTTCTGTC-3′and 5′-CACGCAAATTTCCTTCCACTCG-3′, the primers for *p21* are 5′-CTGGAGACTCTCAGGGTCGAAA-3′ and 5′-CTGCGTAGTTGTGCTGATGT-3′, the primers for *p21* are 5′-CTGGAGACTCTCAGGGTCGAAA-3′ and 5′-CTGGAGACTCTCAGGGTCGAAA-3′, the primers for *GADD45A* are 5′-CTGGAGGAAGTGCTCAGCAAAG-3′ and 5′-AGAGCCACATCTCTGTCGTCGT-3′, the primers for *PAI-1* are 5′-CTCATCAGCCACTGGAAAGGCA-3′ and 5′-GACTCGTGAAGTCAGCCTGAAAC-3′, the primers for *DEC1* are 5′-CTCATCAGCCACTGGAAAGGCA-3′ and 5′-GACTCGTGAAGTCAGCCTGAAAC-3′, the primers for *PUMA* are 5′-CCTGGAGGGTCCTGTACAATCT-3′ and 5′-ACAGTTGCAGCCGTAGTCTTG-3′, the primers for *DR5* are 5′-CCAGGTCGTTGTGAGCTTCT-3′ and 5′-GACTCGTGAAGTCAGCCTGAAAC-3′, and the primers for *GAPDH* are 5′-ACCCACTCCTCCACCTTTGA-3′ and 5′-CTGTTGCTGTAGCCAAATTCG-3′. Calculate the relative quantitative value of the target region using the Ct method with *GAPDH* as the standard.

### 2.8. Protein Stability Assay

Cells were inoculated into 12-well plates at a density of 1 × 10^5^ cells per well and treated with ACY-1215 for 24 h. Protein synthesis was then inhibited by adding 50 µg/mL CHX. For time-course analysis, cells were harvested at 0, 15, 30, 45, and 60 min after CHX treatment. Immunoblotting analysis was used to detect p53 protein expression levels.

### 2.9. Coimmunoprecipitation

Coimmunoprecipitation were performed as described [21]. Cells were washed with ice-cold PBS and lysed in Triton buffer containing protease inhibitors for 15 min at 4 °C. Lysates were sonicated, centrifuged, and incubated with specific antibodies overnight at 4 °C. Agarose beads were then added, followed by 8 h incubation with rotation at 4 °C. Beads were pelleted, washed, resuspended in SDS loading buffer, and boiled for 15 min prior to western blotting.

### 2.10. Mass Spectrometry

Following coimmunoprecipitation, protein G-beads were lysed with 3 × SDT buffer, boiled for 10 min, centrifuged, and the supernatant was resolved by SDS-PAGE. Gel fragments were washed, cut into 1 mm^3^ pieces, and processed for destaining (50 mM NH_4_HCO_3_/50% ACN, 37 °C, 600 rpm), reduction (10 mM DTT, 37 °C, 1 h), and alkylation (55 mM IAM, RT, 40 min in darkness), with intermediate ACN dehydration and NH_4_HCO_3_ washes. Peptides were extracted using ACN gradients (60% and 90% with 0.1% TFA), desalted via C18 spin columns, and vacuum-dried. LC-MS/MS analysis was performed on an Orbitrap Fusion Lumos system (Thermo Fisher Scientific, Waltham, MA, USA) coupled with an EASY-nanoLC 1200 and online nano-electrospray ion source. Raw data were processed using PEAKS Studio v10.6 (Bioinformatics Solutions Inc., Waterloo, ON, Canada).

### 2.11. Statistical Analysis

Quantitative data processing was performed through GraphPad Prism v8 (GraphPad Software Inc., San Diego, CA, USA) with implementation of parametric analysis of variance models, including both single-factor (ANOVA) and multifactorial (two-way ANOVA) comparative approaches. Experimental observations, expressed as arithmetic mean ± standard error of measurement (SEM), originated from triplicate biological experiments conducted under standardized conditions. Statistical significance was defined as * *p* < 0.05.

## 3. Results

### 3.1. ACY-1215 Inhibits Cell Proliferation, Invasion, and Migration, and Induces Apoptosis in HCC Cells

**Figure 1:**
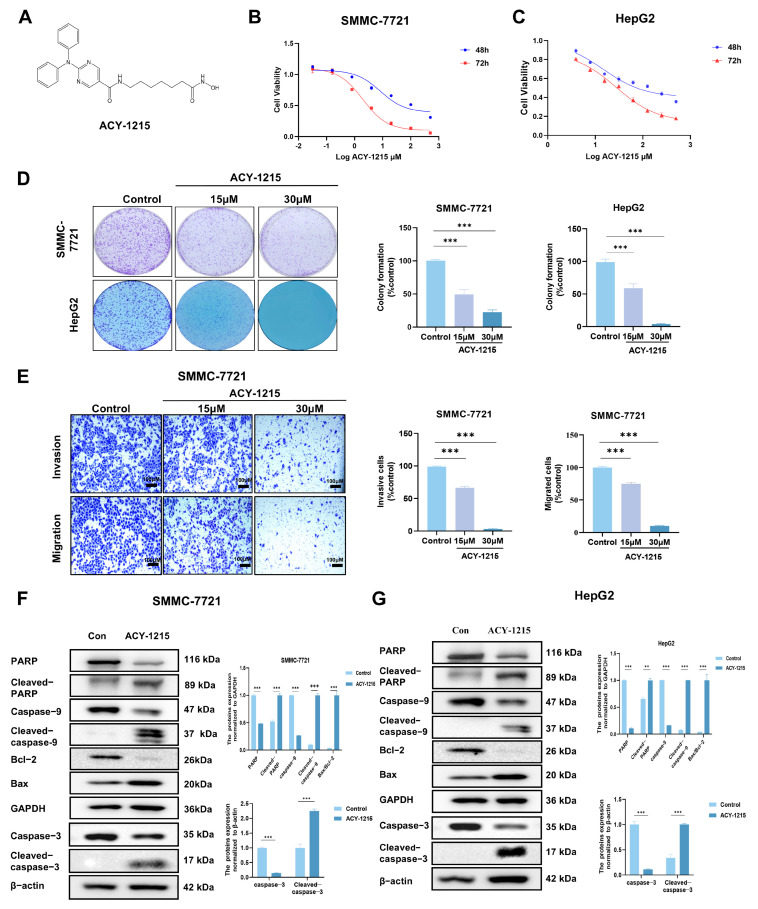
ACY-1215 suppresses HCC cell proliferation and colony formation. (**A**) Chemical structure of ACY-1215. (**B**) Dose- and time-dependent effects of ACY-1215 (4–500 µM) on cell viability in SMMC-7721 and HepG2 cells, assessed by CCK-8 assay after 48 h and 72 h treatment. (**C**) Representative images and quantification of colony formation in SMMC-7721 and HepG2 cells (**D**) treated with ACY-1215 (15 µM and 30 µM) for 14 days. Colonies were fixed with 4% paraformaldehyde, stained with 0.1% crystal violet, and analyzed using ImageJ to calculate the percentage of area covered by colonies (mean of three replicates). (**E**) Migration and invasion capabilities of SMMC-7721 cells following 24 h treatment with ACY-1215, evaluated by Transwell assays. The number of migrating and invading cells was then quantified with Image J. (**F**,**G**) SMMC-7721 and HepG2 cells were treated with ACY-1215 for 24 h. Whole-cell lysates were determined by immunoblotting with the indicated antibodies. Data are shown as the mean ± SEM (n = 3): ** *p* < 0.01 and *** *p* < 0.001.

To investigate the effects of ACY-1215 (Figure 1A) on HCC cell lines, SMMC-7721 and HepG2 cells were treated with different concentrations of the drug for 48 and 72 h. After treatment, cell viability was assessed using the CCK-8 assay. As shown in Figure 1B,C, ACY-1215 significantly inhibited the proliferation of HCC cells. The IC_50_ value was 32.45 ± 1.69 μM at 72 h in the SMMC-7721 cell line and 3.35 ± 0.83 μM at 72 h in the HepG2 cell line. A long-term colony formation assay showed that treatment with ACY-1215 significantly reduced both the number and size of HCC cell colonies in a dose-dependent manner (Figure 1D). Transwell migration and Matrigel invasion assays revealed that ACY-1215-treated HCC cells exhibited reduced migration and invasion (Figure 1E). Immunoblotting was then performed to detect pro-apoptotic proteins (caspase-3, caspase-9, PARP, and Bax) and anti-apoptotic protein Bcl-2. In the SMMC-7721 cell line, treatment with ACY-1215 increased the cleavage levels of caspase-9, caspase-3, PARP, and Bax, decreased the pro-apoptotic protein Bcl-2, and increased the Bax/Bcl-2 ratio (Figure 1F). Similar results were observed in the HepG2 cell line (Figure 1G). These results suggest that the apoptosis-inducing effect of ACY-1215 is mediated through a caspase-dependent pathway. Additionally, the levels of p53 downstream proteins Bax and Bcl-2 were regulated by ACY-1215.

### 3.2. ACY-1215 Enhances the Expression Level of p53 and Inhibits HDAC6-Mediated Deacetylation to Activate the Transcription of Its Downstream Target Genes

**Figure 2:**
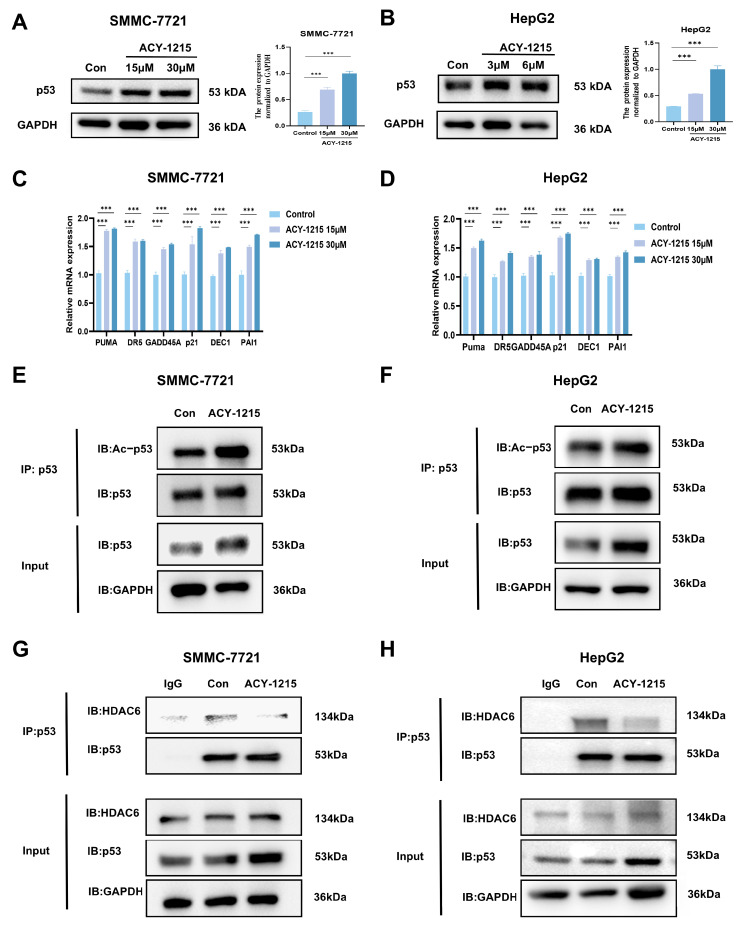
ACY-1215 promotes p53 acetylation by inhibiting HDAC6-mediated deacetylation, stabilizing p53, and activating downstream transcriptional targets. (**A**,**B**) Immunoblot analysis of p53 protein levels in SMMC-7721 and HepG2 cells treated with ACY-1215 for 24 h. Quantification of band intensity (right panel) was performed using ImageJ. (**C**,**D**) RT-qPCR analysis of p53 downstream target gene expression in SMMC-7721 and HepG2 cells following 24 h ACY-1215 treatment. (**E**,**F**) Endogenous p53 acetylation levels in SMMC-7721 and HepG2 cells treated with ACY-1215 for 24 h, assessed by coimmunoprecipitation. (**G**,**H**) Coimmunoprecipitation analysis of HDAC6-p53 interactions in SMMC-7721 and HepG2 cells using IgG control, p53 antibody, and protein G agarose beads. Immunoprecipitates were analyzed by western blotting with anti-HDAC6 and anti-p53 antibodies. Data are presented as mean ± SEM (n = 3); *** *p* < 0.001.

We investigated the effects of ACY-1215 on p53 expression and cellular functions. After ACY-1215 treatment, the p53 protein level showed a trend of up-regulation in both SMMC-7721 and HepG2 HCC cell lines (Figure 2A,B). RT-qPCR experiments showed that the mRNA expression levels of p53 downstream target genes, including apoptosis-related genes (*PUMA* and *DR5*), cell cycle-related genes (*GADD45A* and *p21*), and senescence-related genes (*DEC1* and *PAI1*), were upregulated in ACY-1215-treated HCC cells (Figure 2C,D).

The extent of acetylation modification of p53 significantly affects its activity and function. We further enriched p53 using an immunoprecipitation technique and detected its acetylation status using an acetyl antibody, revealing that the acetylation level of p53 significantly increased after ACY-1215 treatment (Figure 2E,F). As reported in the literature, HDAC6 interacts with p53 and suppresses its transcriptional activity [22]. Given that ACY-1215 functions as a selective inhibitor of HDAC6, we hypothesize that it inhibits the interaction between HDAC6 and p53. In vitro coimmunoprecipitation experiments using endogenous proteins confirmed that ACY-1215 inhibits the interaction between HDAC6 and p53 (Figure 2G,H). These findings suggest that ACY-1215 inhibits HDAC6-mediated deacetylation of p53, resulting in an increased acetylation level of p53 by disrupting the interaction between HDAC6 and p53.

### 3.3. ACY-1215 Inhibits the Ubiquitination Level of p53 and Disrupts the Interaction Between MDM2 and p53

**Figure 3:**
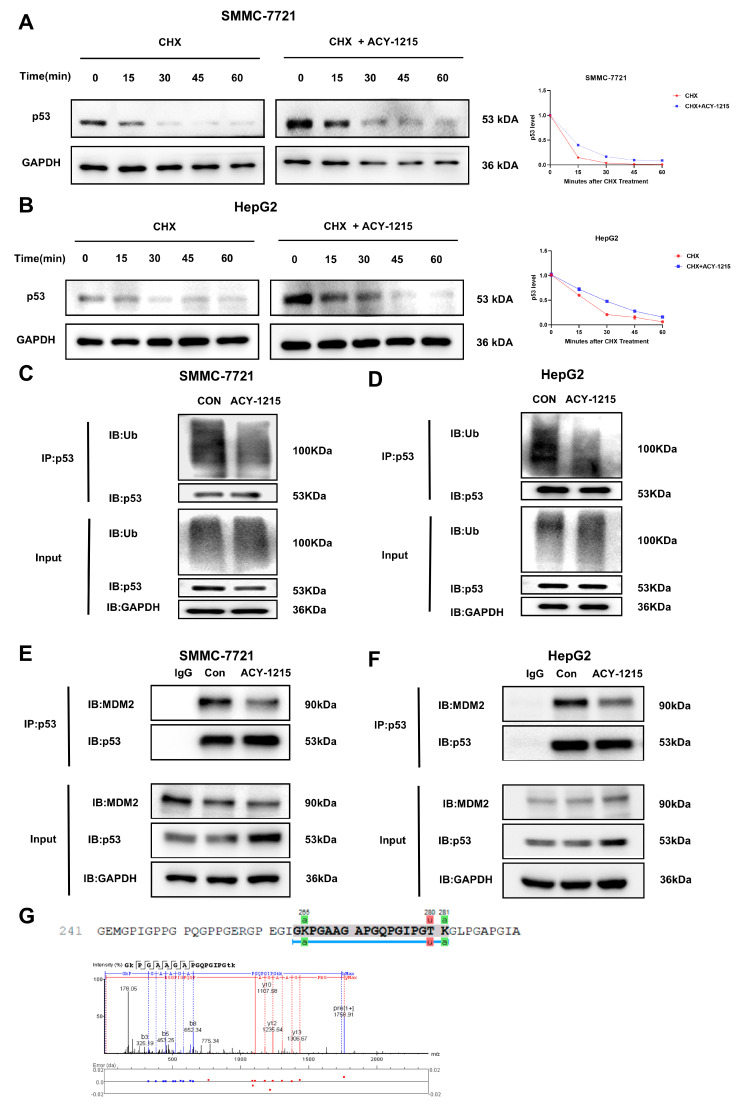
ACY-1215 stabilizes p53 by inhibiting its MDM2-mediated ubiquitination. (**A**,**B**) Western blot analysis of p53 protein levels in SMMC-7721 and HepG2 cells pretreated with or without ACY-1215 for 24 h, followed by CHX treatment for 0–60 min. (**C**,**D**) Coimmunoprecipitation analysis of endogenous p53 ubiquitination in SMMC-7721 and HepG2 cells treated with ACY-1215 for 24 h. (**E**,**F**) Coimmunoprecipitation analysis of MDM2-p53 interactions in SMMC-7721 and HepG2 cells following 24 h ACY-1215 treatment. (**G**) Detection of p53 post-translational modification sites by mass spectrometry analysis. The square brackets in the mass spectra represent a charge. Data are presented as mean ± SEM (n = 3).

The experimental data showed that *p53* transcript levels did not change significantly in HCC cells while its protein expression was significantly down-regulated (Figure S1), suggesting that the low expression of p53 protein may be attributed to the enhanced degradation caused by the reduction of its protein stability. CHX was used to block protein synthesis and the results of CHX experiments showed that ACY-1215 intervention prolonged the half-life and increased the stability of p53 protein (Figure 3A,B). Moreover, we used the proteasome inhibitor MG132, lysosomal pathway inhibitor CQ, and cysteine protease pathway inhibitor E-64 to determine the primary degradation pathway of p53. The results showed that p53 protein expression was increased in the MG132 group in both cell lines, while no significant changes were observed in the other groups (Figure S2), indicating that the ubiquitin-proteasome pathway is the primary degradation pathway of p53. We further investigated whether ACY-1215 affected the levels of ubiquitination-mediated proteasomal degradation of p53. We precipitated endogenous and labeled p53 from SMMC-7721 and HepG2 cells and detected its ubiquitinated form to confirm the effect of ACY-1215. Similarly, ACY-1215 caused a significant reduction in p53 ubiquitination levels (Figure 3C,D). MDM2 serves as the major E3 ubiquitin ligase regulating p53 through direct binding, catalyzing its polyubiquitination and targeting it for proteasome-mediated degradation [23]. The experimental results also showed that ACY-1215 disrupted the interaction between MDM2 and p53 (Figure 3E,F), leading to a decrease in p53 ubiquitination levels and an increase in protein stability. Based on the results above, we found that the stability of the p53 protein is closely related to its acetylation and ubiquitination modifications. Next, we performed mass spectrometry analysis to detect the specific sites of post-translational modifications of the p53 protein. According to the results of mass spectrometry, the p53 protein has adjacent modifications at the K280 (ubiquitination, u) and K281 (acetylation, a) sites. This spatial proximity suggests that there may be a dynamic competition or mutual regulation mechanism between the two modifications (Figure 3G).

## 4. Discussion

Hepatocellular carcinoma, characterized by a low TP53 mutation rate but frequent functional inactivation of wt-p53 through dysregulated post-translational modifications (PTMs), presents a critical challenge in oncology [24]. PTMs, such as ubiquitination and acetylation, dynamically govern p53 stability, localization, and transcriptional activity, playing a central role in tumorigenesis [25]. This study demonstrates that ACY-1215, a selective HDAC6 inhibitor, restores p53 function by dual targeting of HDAC6 and MDM2—key regulators of PTMs. ACY-1215 inhibits HDAC6-mediated deacetylation, restoring p53’s DNA-binding capacity and transcriptional activity, while concurrently blocking MDM2-dependent ubiquitination to extend p53’s half-life. This dual regulatory mechanism not only elucidates the molecular basis of ACY-1215’s antitumor efficacy but also reveals a potential synergistic interplay between acetylation and ubiquitination modifications. A key hypothesis is that acetylation may act through steric hindrance to disrupt the binding interface of p53 with MDM2, its primary E3 ubiquitin ligase, thereby blocking ubiquitin conjugation and subsequent proteasomal degradation. This proposed spatial competition underscores a dynamic crosstalk between post-translational modifications, where acetylation could stabilize p53 by antagonizing its ubiquitination-dependent turnover. Integrating mass spectrometry data, we propose that ACY-1215 stabilizes acetylation at lysine residues K281, inducing conformational rearrangements in p53 that reduce spatial accessibility of the MDM2-binding domain located within the N-terminal transactivation domain. This structural shift establishes an “acetylation-ubiquitination repulsion” mechanism. This hypothesis aligns with prior mechanistic studies demonstrating that acetylation competitively impedes ubiquitin chain assembly through steric hindrance. The mechanistic interplay between acetylation and ubiquitination is exemplified in colon cancer models, where acetylation of the K381/382 residues disrupts HDAC6-p53 complex formation and enhances transcriptional activity by unmasking p53’s DNA-binding domain, as demonstrated in prior studies [26]. This PTM-based molecular mutual exclusion phenomenon may be universal. For instance, Small Ubiquitin-like Modification (SUMOylation) at the K386 site of p53 creates steric hindrance due to the bulky SUMO conjugate, which physically obstructs access to adjacent lysine residues (K373 and K382), thereby impeding p300-mediated acetylation [27]. These findings suggest that there is a complex “modification code” regulatory system in the PTM network.

ACY-1215 demonstrates therapeutic superiority over conventional therapies through its dual action. In contrast to pan-HDAC inhibitors (e.g., Vorinostat) associated with dose-limiting hematotoxicity, this HDAC6-selective inhibitor employs a rationally designed pharmacophore incorporating a urea linker and a hydroxamic acid-based zinc-binding motif to achieve target-specific inhibition [28]. In addition, compared with MDM2 small molecule inhibitors, ACY-1215 also demonstrated unique therapeutic advantages by regulating p53 stability through a dual mechanism. Conventional MDM2 inhibitors, exemplified by Nutlin-3, suppress p53 ubiquitination-dependent degradation through competitive occupation of MDM2’s p53-binding pocket, thereby preventing MDM2-p53 complex formation, as established in prior mechanistic studies [29]. However, clinical studies have shown that long-term use of MDM2 inhibitors is prone to induce MDM2 gene amplification or protein overexpression, leading to acquired resistance [30]. In contrast, ACY-1215 not only inhibited MDM2-mediated p53 ubiquitination degradation but also improved its protein stability by enhancing p53 acetylation modification through targeting both HDAC6 and MDM2 regulatory nodes. Moreover, this dual mechanism of action may be more effective in overcoming MDM2 overexpression-mediated drug resistance. On the one hand, the acetylation modification induces a conformational change in p53 that reduces its binding affinity to MDM2, while on the other hand, the high acetylation state promotes p53 nuclear translocation away from the cytoplasmic compartment where MDM2 mainly exerts its function.

Studies have demonstrated that HDAC6 can influence tumor cell migration by modifying substrates such as α-tubulin, in addition to regulating p53 [31]. This may partially account for the anti-metastatic effects of ACY-1215 observed in in vitro models. High-throughput mass spectrometry analysis revealed that p53 exists in various post-translational modifications, including sumoylation and phosphorylation. Studies have shown that these modifications affect the activity and stability of the p53 protein [27,32]. Future studies should employ modification site mutant experiments to clarify the specificity of these interactions.

This study demonstrates that ACY-1215 exerts significant anti-tumor effects in HCC by targeting HDAC6/MDM2 to modulate p53 post-translational modification. However, the synergistic mechanism of HDAC6 and MDM2 in p53-dependent tumors requires further exploration. Do HDAC6 and MDM2 directly collaborate to regulate p53 through a functional complex? Our current results suggest that both may indirectly affect p53 stability through spatial site blocking effects, or they may form functional complexes that directly collaborate to regulate it. Specifically, HDAC6-mediated deacetylation of p53 at K281 may expose its C-terminal ubiquitination site, thereby promoting MDM2-mediated proteasomal degradation. However, structural biological evidence is still needed to confirm whether the two form a stable ternary complex with p53. Additionally, clinical phase I/II trials have shown that ACY-1215, used alone or in combination with proteasome inhibitors (e.g., bortezomib), significantly improves progression-free survival (PFS) in patients with relapsed/refractory multiple myeloma (MM) and is well tolerated. The main adverse effects are mild fatigue, nausea, and thrombocytopenia; cardiotoxicity, commonly associated with conventional HDAC inhibitors, was not observed [18,33].

However, this study has several limitations. First, the exact mechanism of HDAC6 and MDM2 synergy in regulating p53 remains unclear. Second, while ACY-1215 shows clinical efficacy in multiple myeloma, its anti-tumor effects and safety in HCC patients need confirmation in dedicated trials, for example, further evaluation of long-term toxicity and optimal treatment strategies in large-scale clinical phase III trials.

## 5. Conclusions

**Figure 4:**
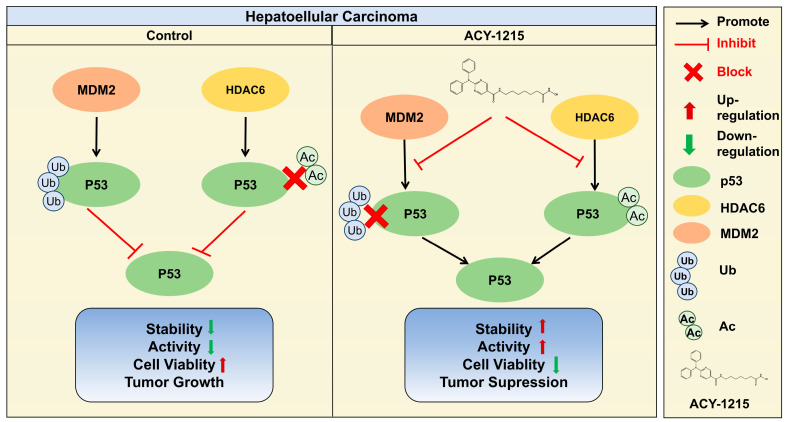
Graphical abstract: schematic representation of the anti-HCC effect of ACY-1215. As shown, ACY-1215 inhibited MDM2-mediated ubiquitination degradation of p53 and inhibited HDAC6-mediated deacetylation, resulting in increased stability of p53 and tumor cell suppression.

ACY-1215 suppresses hepatocellular carcinoma progression by inhibiting proliferation, migration, and invasion, and triggering caspase-dependent apoptosis. It enhances p53 stability through HDAC6 inhibition, promoting acetylation while blocking MDM2-mediated ubiquitination, thereby activating tumor-suppressive pathways (Figure 4). These findings highlight ACY-1215’s dual-action therapeutic potential in HCC via post-translational modulation of p53.

## Data Availability

Data are contained within the article.

## Supplementary Materials

The following supporting information can be downloaded at: https://www.mdpi.com/article/10.3390/cimb47050338/s1.
