# Supplementary material for: Mechanistic Insights into the Anti-Hepatocellular Carcinoma Effects of ACY-1215: p53 Acetylation and Ubiquitination Regulation
Source: Curr Issues Mol Biol. 2025 May 8;47(5):338. doi: 10.3390/cimb47050338 (PMC12110493; doi:10.3390/cimb47050338)
Supplement: Supplementary file 1 [file cimb-47-00338-s001.zip › cimb-3600565-supplementary.pdf]

S1

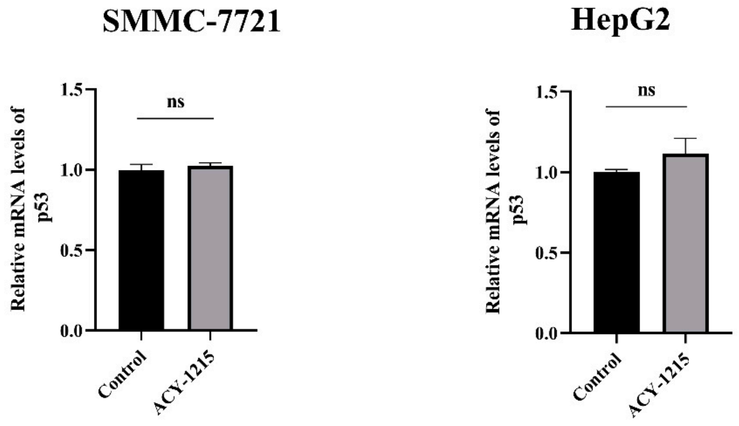

Figure S1 ACY-1215 treatment did not affect p53 transcription levels. SMMC-7721 cells and HepG2 cells were treated with ACY-1215 for 24 h. RT-qPCR assay was used to detect the effect of ACY-1215 on p53 gene. Data represent the mean ± SEM (n=3): ns p>0.05(no significant difference)

S2

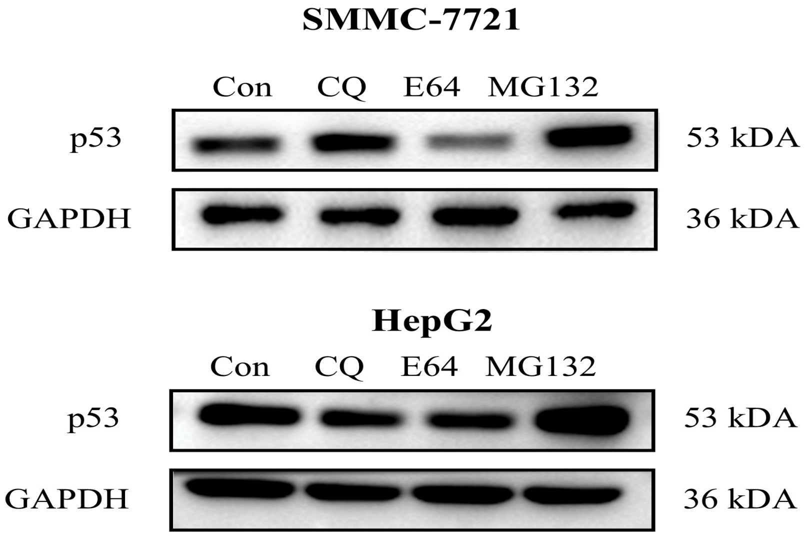

Figure S2 The most important pathway for p53 degradation is the ubiquitin proteasome pathway.SMMC-7721 and HepG2 cells were intervened with proteasome inhibitor MG132, lysosomal pathway inhibitor CQ, and cysteine protease pathway inhibitor E64 for 24h, and the protein expression level of p53 was detected by immunoblotting.
